# A 117-year retrospective analysis of Pennsylvania tick community dynamics

**DOI:** 10.1186/s13071-019-3451-6

**Published:** 2019-04-29

**Authors:** Damie Pak, Steven B. Jacobs, Joyce M. Sakamoto

**Affiliations:** 1 Department of Biology, Pennsylvania State University, W-234A, Millennium Science Complex, University Park, PA 16802 USA; 2 Department of Entomology, Pennsylvania State University, 501 ASI Building, University Park, PA 16802 USA; 3 Department of Entomology, Pennsylvania State University, W-104 Millennium Science Complex, University Park, PA 16802 USA

**Keywords:** Ticks, Passive surveillance, Museum collections, Community composition

## Abstract

**Background:**

Tick-borne diseases have been increasing at the local, national, and global levels. Researchers studying ticks and tick-borne diseases need a thorough knowledge of the pathogens, vectors, and epidemiology of disease spread. Both active and passive surveillance approaches are typically used to estimate tick population size and risk of tick encounter. Our data consists of a composite of active and long-term passive surveillance, which has provided insight into spatial variability and temporal dynamics of ectoparasite communities and identified rarer tick species. We present a retrospective analysis on compiled data of ticks from Pennsylvania over the last 117 years.

**Methods:**

We compiled data from ticks collected during tick surveillance research, and from citizen-based submissions. The majority of the specimens were submitted by citizens. However, a subset of the data was collected through active methods (flagging or dragging, or removal of ticks from wildlife). We analyzed all data from 1900–2017 for tick community composition, host associations, and spatio-temporal dynamics.

**Results:**

In total there were 4491 submission lots consisting of 7132 tick specimens. Twenty-four different species were identified, with the large proportion of submissions represented by five tick species. We observed a shift in tick community composition in which the dominant species of tick (*Ixodes cookei*) was overtaken in abundance by *Dermacentor variabilis* in the early 1990s and then replaced in abundance by *I. scapularis*. We analyzed host data and identified overlaps in host range amongst tick species.

**Conclusions:**

We highlight the importance of long-term passive tick surveillance in investigating the ecology of both common and rare tick species. Information on the geographical distribution, host-association, and seasonality of the tick community can help researchers and health-officials to identify high-risk areas.

**Electronic supplementary material:**

The online version of this article (10.1186/s13071-019-3451-6) contains supplementary material, which is available to authorized users.

## Background

**Fig. 1:**
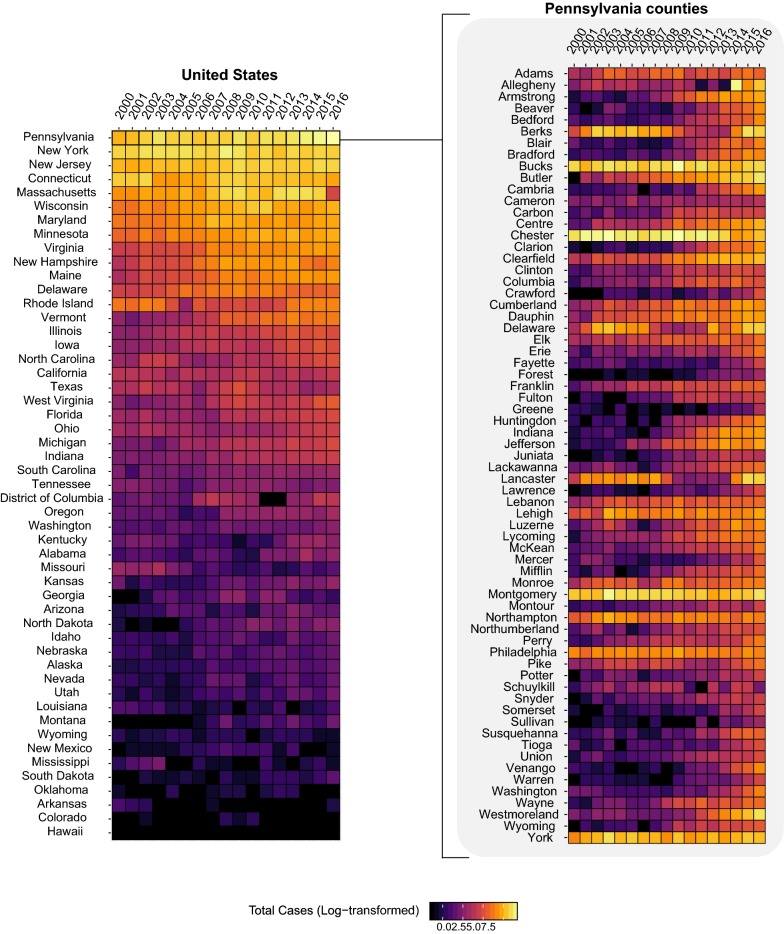
Annual reported cases of Lyme disease. By state from 2006–2017 (left) and by counties in Pennsylvania from 2006–2017 (right). Public data from the Centers for Disease Control and Prevention

The Centers for Disease Control and Prevention reported a 3.5× increase in vector-borne diseases in the USA between 2004–2016, with 76.5% of cases caused by tick-borne pathogens [1]. The increase in tick-borne disease is attributed to climate change, land use changes, and expanding geographical ranges for several important endemic tick species, posing novel risks to local communities [2, 3]. Although there are many tick-borne pathogens, the vast majority of tick-borne disease cases are caused by *Borrelia burgdorferi* [1, 4], the main etiological agent of Lyme disease in the USA. Pennsylvania has had the highest number of total Lyme disease cases since 2000, with increasing numbers of annual cases across several counties (Fig. 1).

Surveillance data collected over multiple decades may reveal spatio-temporal changes in ectoparasite communities [5]. Data such as spatial distribution and occurrence of both abundant and rare species of ticks can be correlated with land use (e.g. habitat loss, fragmentation, management), fluctuating environmental conditions, or changes in human or animal behavior (e.g. encroachment may bring reservoir hosts in closer proximity) [3, 6]. Long-term surveillance data can also reveal shifts in the temporal dynamics of tick populations and communities [2]. Although the seasonality of known tick species has been described, year-to-year distribution of tick species may be influenced by inter-annual variability (e.g. local climate) and biotic factors (e.g. local reservoir species abundance). These data can be useful for developing predictive models that accurately measure the risks of tick-borne zoonotic agents.

We present a retrospective analysis of tick collection data in Pennsylvania from the early 1900s to June of 2017. Some of the data prior to 1968 had been published in a progress report on ticks from Pennsylvania but were presented in a format that included anecdotes and overall percentages rather than raw number breakdowns by species [7]. We revisit these specimens and utilize the raw data from both these time periods (1900–1967 and 1968–June 2017) to identify shifts in tick community composition, phenological patterns, and host associations. We used our database to map the distribution of major tick species at the county level, investigate tick community spatiotemporal dynamics, and explore host associations by tick species.

## Methods

### Study locations

The state of Pennsylvania (PA) is located in the Northeast/Mid-Atlantic region of the USA (State Center: 40.9699889, −77.7278831 [8]. Climate in Pennsylvania is variable by location, but broadly classified as a continental type with warm, humid summers (mean temperature ranges of 17.7–23.33°C, [9]. The majority of Pennsylvania’s land-use is dedicated to agriculture (both croplands and pastures), forestland, with some dense urban areas [9, 10]. There have been significant changes in the human populations of PA from 1960 to 2010, but a large proportion of the PA population has remained heavily clustered around Philadelphia and Pittsburgh, which are located in south-eastern and in south-western PA respectively (Additional file 1: Figure S1).

### Submissions

The PSU Frost Entomological Museum (‘Frost Museum’) houses arthropod samples collected by researchers, teaching collections, and samples submitted by the public for identification. We present our analysis of the tick specimens from 1900 to June 2017, although some Frost Museum collections date as far back as the late 1800s. Because tick samples were submitted to the Department of Entomology or the Frost Museum over a period of 117 years, they represent multiple collection/submission periods (early 1900–1959; 1960–1969; 1970–1988; Tick Research Lab (TRL) submissions from 1990 to 1993 and 1995–present). Two public campaigns account for the majority of the citizen-submitted specimens. The first campaign (between 1963–1967) was conducted by Dr Robert Snetsinger. He enlisted the help of the public through advertisements in radio, television, and newspapers to obtain 700 specimens [7]. Additionally, he utilized active surveillance methods to collect approximately 500 ticks using a combination of dragging, sweeping, live animal trapping, and roadkill examinations of mammals and birds to assess tick abundance in localized areas [7]. A second funded campaign dedicated to estimating tick abundance and species diversity was launched by Steven B. Jacobs (second author) from 1990 to 1993 (TRL), in which he cataloged, identified, and labeled each specimen. Specimens were accompanied by additional data: date of tick discovery, location and vegetation type of tick encounter, and host species.

Data from both campaigns and subsequent submissions were combined into a single dataset for our analyses. For analysis on the distribution of tick species over time, we used total tick counts. For quantifying host association, however, we used “submission” number which we defined as a vial or lot containing one or more ticks. We chose to use this more conservative measure rather than total specimen count to avoid skews in abundance by hosts. For example, a submission lot of 1 tick *versus* 50 ticks from a host were both classified as “one submission”.

While most specimens were collected within state boundaries, a few were declared from people either visiting or returning from visiting other states. Tick specimens identified as species that are not commonly found in Pennsylvania were later discovered to have been imported from other states/countries or found on exotic animals. Non-PA data were excluded from state-wide analyses, but were included in Additional file 2: Tables S1 and Additional file 3: Table S2).

### Identification

Ticks were morphologically identified to species and life stage using taxonomic keys for Argasidae, Ixodidae east of the Mississippi, *Dermacentor*, nymphal *Ixodes*, and nymphs of *Amblyomma* [11–17]. Species-level identification is crucial since at least 3 *Dermacentor* species, 3 species of *Amblyomma*, and 9 different *Ixodes* species have been reported in Pennsylvania. If diagnostic characters were missing due to damage to the specimen, the next level of taxonomic identification was used (e.g. samples with missing mouthparts that were clearly Prostriata were identified as “*Ixodes* spp.”). In a few cases, samples were not identified beyond “tick” and were designated “Ixodidae” for hard ticks or “Argasidae” for soft ticks. Unusual specimens or those that were difficult to identify were sent to the National Tick Collection, Georgia Southern University for confirmation (by Dr James Oliver at the time of confirmation).

### Spatial distribution

We focused on the geographical distribution of the five most abundant species of significant public health and veterinary importance: *Amblyomma americanum* (Linnaeus), *Dermacentor variabilis* (Say), *Ixodes cookei* (Packard), *Ixodes scapularis* (Say), and *Rhipicephalus sanguineus* (Latreille). Working on the assumption that counties with higher populations would submit more specimens than less populated counties, we estimated the prevalence rate (the total numbers of individual ticks per 100,000 people). This was done by adjusting the total tick count of each species by the countyʼs total population. We looked at relevant time periods during the surveillance programme: 1960–1969; 1990–1999; 2000–2009; and 2010–2018. For each time period, we used the United States Census data for 1960, 1990, 2000 and 2010, respectively, to calculate the tick prevalence rate (Additional file 1: Figure S1) [18–20]

### Temporal analysis

We used the annual sum of all individual tick specimens to investigate how annual submission rates changed over time. We then analyzed the temporal dynamics of the five most abundant taxa (*A. americanum*, *D. variabilis*, *I. cookei*, *I. scapularis* and *R. sanguineus*). We did not evaluate total counts by year as these varied drastically due to the active campaigning for citizen submissions or the introduction of identification fees. Therefore, we looked at the proportional contribution of each species to the annual summed counts of all the five major species. To detect if there have been any monotonic trends (i.e. gradual shifts in abundance), we ran a non-parametric, two-sided Mann-Kendall trend test on the yearly proportion of each of the species between 1900–2017.

We investigated the seasonal distribution of the tick community by analyzing the monthly frequency of submissions. Citizen submissions were to include the date of discovery, but for specimens that lacked these, we used the date that a given submission was received. We analyzed the seasonal distribution of motile life stages (larvae, nymphs and adults) for the five most abundant taxa. The proportions were calculated by comparing the monthly abundance of each life stage (larvae, nymphs and adults) to the cumulative sum of all stages.

### Host associations

Host information (combined by family, except for dog, cat, human and groundhog) was available for many of the tick submissions. Host data were classified as either domestic or wildlife. We analyzed the host-tick data by summing the total submissions by both the tick species and the host group. We constructed a circular network map to visualize the relationships between tick species and hosts. All host association analyses were done with R (Version 3.4.13) with the packages *Kendall* for the Mann-Kendall test and the *circlize* package for chord diagrams of host association mapping [21, 22].

## Results

A total of 4491 submission lots consisting of 7132 tick specimens across 23 species were identified (Table 1). Five species of ticks accounted for the majority (91%) of the total number of tick specimens: *Dermacentor variabilis* (*n *= 3172); *Ixodes scapularis* (*n* = 1899); *Ixodes cookei* (*n* = 897); *Rhipicephalus sanguineus* (*n *= 332); and *Amblyomma americanum* (*n* = 196). Other tick species that had at least 100 specimens were *Dermacentor albipictus* (Packard) (*n* = 107), *Ixodes dentatus* (Marx) (*n* = 120), and *Ixodes texanus* (Banks) (*n* = 111). The remaining ticks had less than 100 specimens/species and included both hard and soft tick species.

**Table 1:** The total submissions to the PSU Department of Entomology/Frost Entomological Museum from 1900 to 2017. Generic names that have been changed since the submission date are shown in parentheses

Species	No. of submission lots	No of specimens
*Amblyomma americanum*	183	196
*Amblyomma cajennense*	1	1
*Amblyomma dissimile*	8	8
*Amblyomma longirostre*	1	1
*Amblyomma maculatum*	5	5
*Amblyomma* (*Aponomma*) *transversale*	2	2
*Amblyomma* sp.	2	2
*Argas cooleyi*	2	2
*Argas persicus*	3	3
*Carios* (*Ornithodoros*) *kelleyi*	18	18
*Dermacentor albipictus*	81	107
*Dermacentor andersoni*	1	1
*Dermacentor variabilis*	1498	3172
*Haemaphysalis chordeilis*	1	1
*Haemaphysalis leporispalustris*	38	78
*Ixodes affinis*	1	1
*Ixodes angustus*	39	39
*Ixodes cookei*	661	897
*Ixodes dentatus*	115	120
*Ixodes marxi*	46	48
*Ixodes muris*	6	6
*Ixodes scapularis*	1395	1899
*Ixodes texanus*	16	111
*Ixodes* sp.	79	80
*Rhipicephalus sanguineus*	287	332
*Rhipicephalus* sp.	1	1
Not identified	1	1
Total	4491	7132

### Spatial analysis

**Fig. 2:**
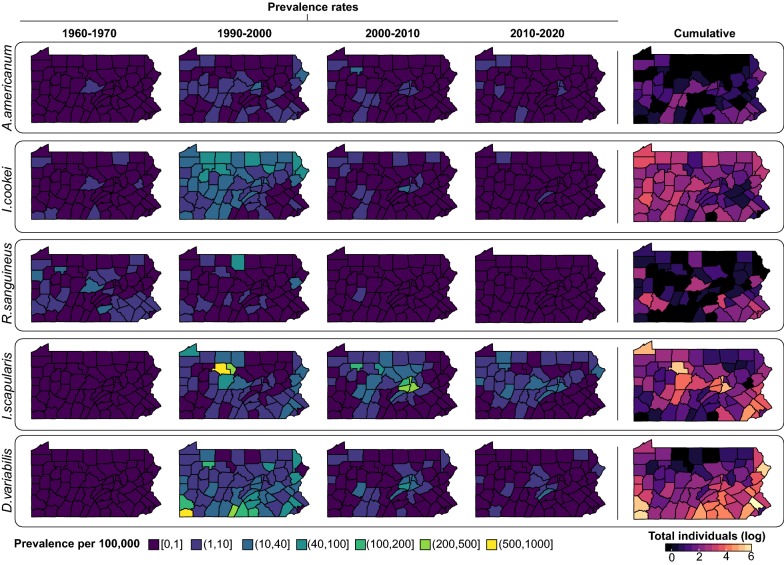
Distribution of the five most abundant tick species across Pennsylvania over time. Prevalence rates (tick counts per 100,000 population, left) represent tick abundance adjusted by county population for time periods 1960–1969, 1990–1999, 2000–2009 and 2010–2018. Cumulative counts of ticks by species shown on the right

Ticks were submitted from all 67 counties in Pennsylvania (Additional file 4: Figure S2). We hypothesized that more tick submissions would come from areas with higher human populations. As expected, tick submissions were heavily clustered around Allegheny and Philadelphia Counties, where Pittsburgh and Philadelphia are located, respectively. When we adjusted the total tick count by county population-decade, we found higher prevalence rates in less populated counties. For example, in 1990–2000, the highest prevalence rates of *I. scapularis* submissions were from Elk County (870 individuals per 100,000 population). Neighboring Forest and Cameron counties also had high submissions of *I. scapularis* with 116.64 and 589.97 individuals per 100,000 respectively (Fig. 2).

*Dermacentor variabilis* distribution was largely localized to the southern parts of the state. From 1990 to 2000, the highest proportion of *D. variabilis* submissions came from Greene County, the most southeastern county of Pennsylvania (865.45 submissions per 100,000). Other southern counties with high tick loads per capita included Fulton County (350.60 per 100,000) and Franklin County (117.26 per 100,000). *Ixodes cookei* was more evenly distributed throughout Pennsylvania, although like *I. scapularis*, it was more highly abundant in the northern counties. In 1990–2000, Forest County had the highest prevalence rates of *Ixodes cookei* with 80.87 per 100,000. *R. sanguineus* and *A. americanum* had very few submissions and their distribution was mostly scattered across Pennsylvania (Fig. 2).

For the tick species with less than 150 submissions across 1900 to 2017, we aggregated the submissions by genus. Multiple species within the genera *Ixodes* and *Dermacentor* were widely distributed across Pennsylvania (*I. scapularis*, *I. cookei*, *D. andersoni* and *D. albipictus*) (Additional file 4: Figure S2). Other species in the genera *Amblyomma*, *Argas*, *Carios* (*Ornithodoros*) and *Haemaphysalis* were not as widely distributed, possibly because these species are not commonly encountered or because the specimens were introduced from their native geographical ranges (Additional file 4: Figure S2, Additional file 5: Figure S3).

### Temporal shifts in species abundance

**Fig. 3:**
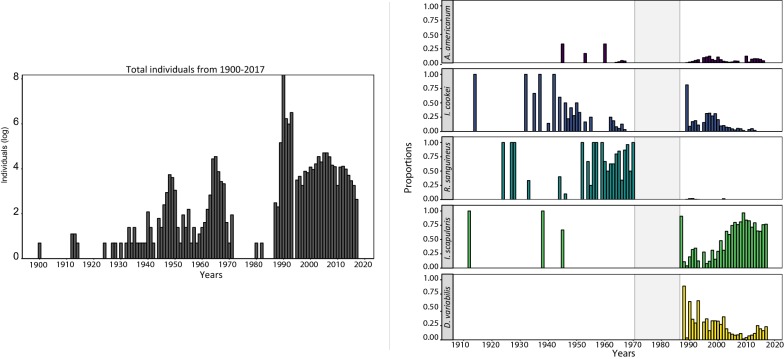
Annual submissions of tick specimens by year. On the left is the annual sum of all tick counts (log-transformed) from 1900 to 2017. On the right are the proportional contributions of the five major tick species to the total tick counts (1900–2017). The grey shaded area represents periods where there were few or no tick submissions from the top five most abundant taxa

Prior to the 1990s, the majority of the tick submissions were identified as *I. cookei* and *R. sanguineus* (Fig. 3). The spike in the number of submissions in 1990 was largely due to *D. variabilis*, but gradually, *I. scapularis* became the dominant taxon submitted. Results from the Mann-Kendall test supports these observations with an upward trend in the *I. scapularis* counts (*tau* = 0.288, *P* = 0.02) and a significant downward trend in *D. variabilis* (*tau* = −0.408, *P* = 0.002). The Mann-Kendall also indicate that the proportional contributions of *I. cookei* (*tau* = −0.607, *P* < 0.001) and *R. sanguineus* (*tau* = −0.377, *P* = 0.005) to the total count have also significantly shifted over a century.

### Seasonality

**Fig. 4:**
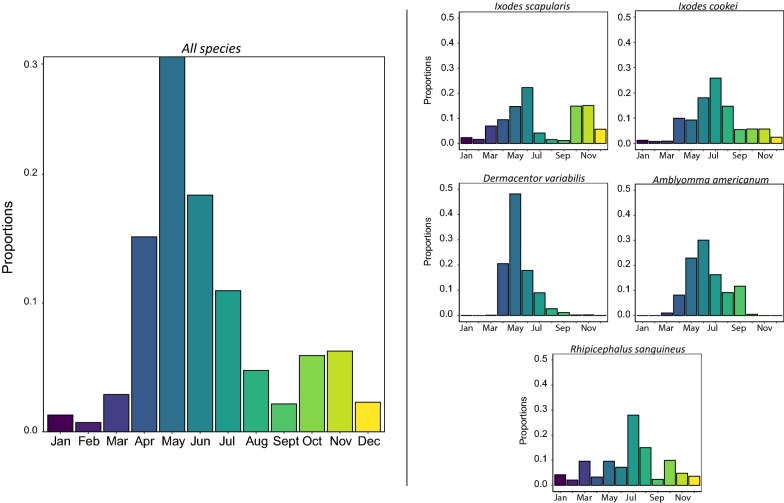
Seasonal distribution of tick submissions over time. On the left is the total proportion of tick specimens received at different months of the years from 1900 to 2017. On the right are the proportional seasonal abundances of each of the five major tick species (1900–2017)

Overall, we find that the majority of tick specimens were received in the months between April and July with the highest proportion of tick submissions in May (Fig. 4). Submissions of *D. variabilis*, *A. americanum*, *I. cookei* and *R. sanguineus* were most abundant during the period between May and July. *Dermacentor variabilis* and *A. americanum* were most abundant from March to October. *Ixodes cookei* and *R. sanguineus* samples were submitted throughout the year, with peak abundance in June. Samples of *I. scapularis* were also submitted year-round, but the peak abundances were bimodally distributed with a large peak between May-June and a second peak between October-November.

**Fig. 5:**
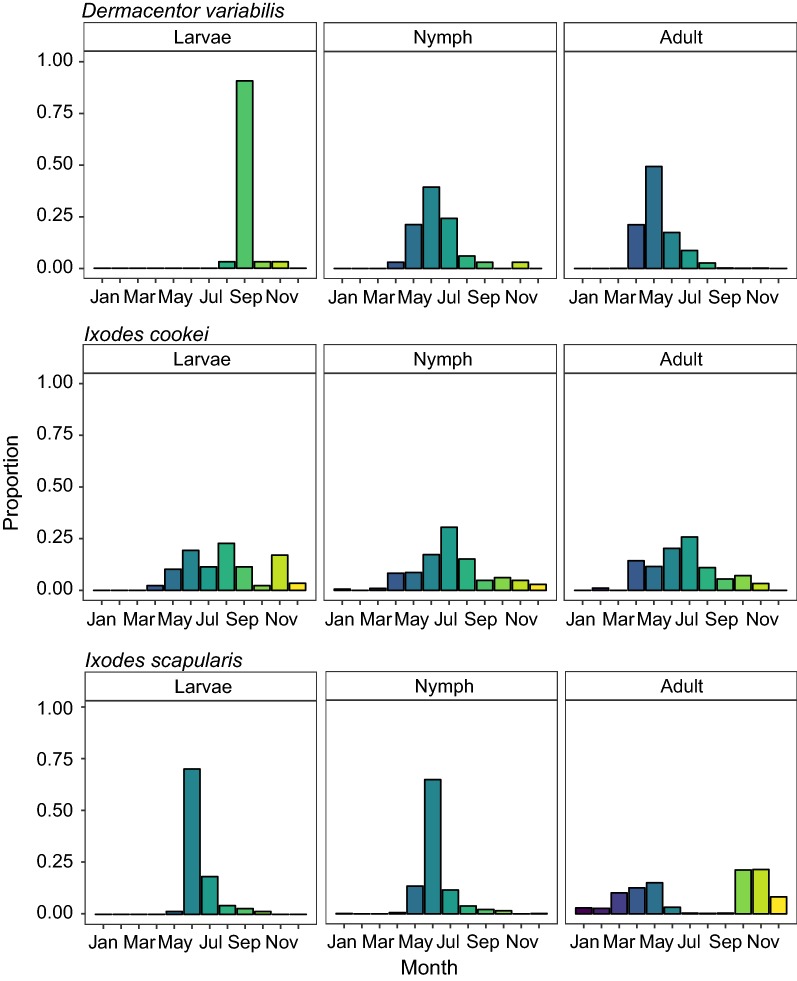
The seasonal distribution of *D. variabilis*, *I. cookei* and *I. scapularis* specimens by life stages from 1900 to 2017. The proportion was calculated by comparing the monthly abundance of each life stage (larvae, nymphs and adults) to the cumulative sum of all stages by species

We compared seasonal distribution by lifestage for 6233 of the 7132 total of tick specimens (Fig. 5). Four percent of total submissions were larvae (*n* = 237), 20% were nymphs (*n* = 1271), and 75% of the submissions were adults (*n* = 4725). Of the *D. variabilis* submissions, there was a total of 32 larvae, 33 nymphs and 3059 adults from 1960 to 2017 (Fig. 5), with a unimodal distribution peaking around June.

Prior to 1990 *I. scapularis* were very rare so we have only shown their lifestage-specific seasonal abundance since 1990. Overall, the nymphal and larval submissions showed a unimodal pattern with the highest proportion of submissions received in June. For the adult submissions, there were prominent bimodal peaks in May and October with similar proportions of submissions received in both seasons.

The majority of *I. cookei* submissions were nymphs (*n *= 521), followed by adults (*n* = 182) and larvae (*n* = 88). The submission patterns indicate that *I. cookei* specimens can be encountered year-round, but that nymphs were the most commonly encountered lifestage. Across all lifestages, we see that the distributions are unimodal with peaks in early summer between May and June.

There were too few lifestage submissions for *A. americanum* and *R. sanguineus* to make sufficient comparisons with the seasonality of these species.

### Host association

**Fig. 6:**
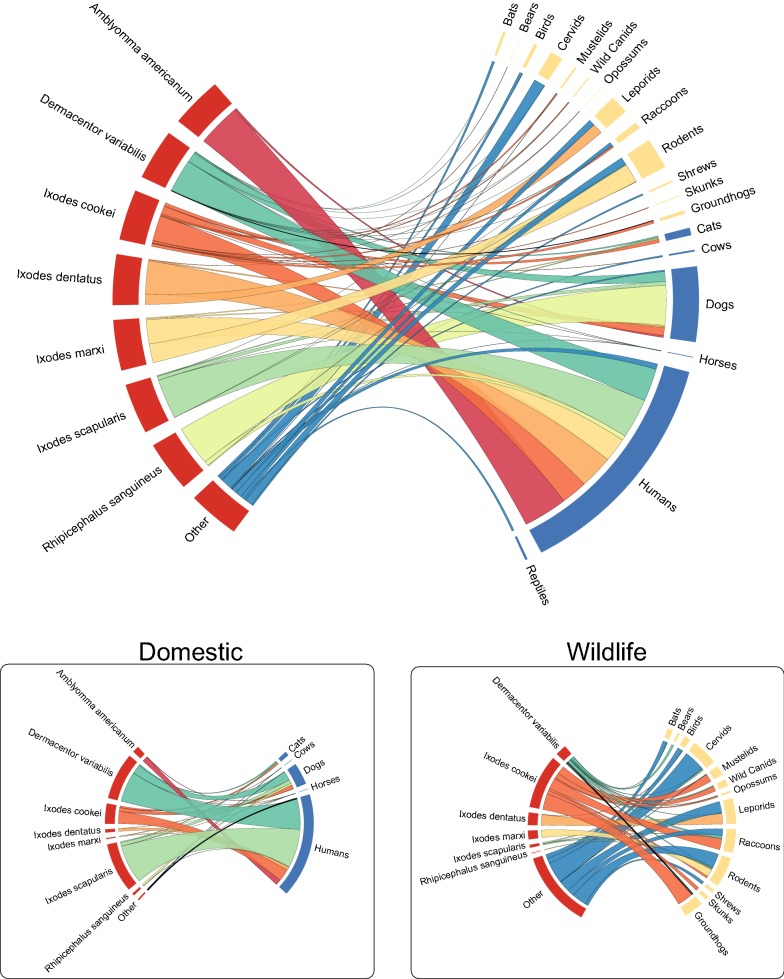
Chord diagram representing associations between tick species and vertebrate hosts parasitized. Submissions (not counts) were used to quantify host association. We chose submissions over counts to avoid skews in abundance by hosts. The wider the chord, the more submissions exist for any given tick species-to-host

One of the assumptions about passive surveillance is that there is an inherent bias toward humans as the hosts, particularly since most specimens submitted by the humans from themselves, their pets, or other domestic animals. By far the majority of submissions were associated with humans and their domestic animals and this reflects the fact that many of the specimens in our collection were submitted by people on themselves or their pets (Fig. 6). Of 4491 submissions from PA, there were 2662 attached to humans, 666 associated with cats or dogs, 20 from other domestic animals, and 168 submissions pooled from multiple hosts (mixed). There were 11 additional submissions found on various exotic animals. There were 689 submissions for which there was no host record or the ticks were not attached to a host. The remaining 275 were found on various wildlife.

## Discussion

Our data is unique in that it contains details about tick community composition and spatio-temporal dynamics from Pennsylvania over a 117-year period. Subsets of our data had been reported as percentages or combined with data from other museums and literature reviews to estimate the distribution of one or more tick species across the state of Pennsylvania [7, 23]. To our knowledge, this is the first time that these data have been compiled in their entirety and analyzed in this format. We were able to detect seasonality, shifts in tick community composition, and host associations that have not been well-documented in a quantitative manner. The seasonality data for the five most abundant tick species inferred by our passive surveillance data is consistent with previous records of seasonality described by other researchers [24–27], demonstrating that these types of passive data contain biologically meaningful signal.

### Shifts in tick community composition

In the 1960s, PA tick communities consisted predominantly of three species: *I. cookei*, *D. variabilis*, and *R. sanguineus* (Fig. 2). The most abundant species at that time, *I. cookei*, often referred to as a groundhog tick, is actually a broad- host tick feeding principally on medium mammals, although humans and dogs will also be parasitized [28]. The second most abundant species*, D. variabilis*, was widely distributed and eventually became the dominant species submitted over *I. cookei* in the 1990s. By 1991 *D. variabilis* had been identified from all but 4 counties (Fig. 2) [23]. After 1995, *D. variabilis* annual submission rates declined as *I. scapularis* submission rates increased (Fig. 2). Because there were gaps in submission numbers for certain years, we cannot say for certain why there were shifts in the abundance of these species.

Although we cannot directly infer a causal negative relationship between *D. variabilis* and *I. cookei* with *I. scapularis*, this pattern was also observed in neighboring Ohio. The passive surveillance programme run by Ohio Department of Health (started in 1978) did not detect *I. scapularis* (=formerly *I. dammini*) until 1989 [29]. At that time, the dominant species were *D. variabilis* (~97% of submissions) and *I. cookei* (1.2%) [24]. Between 1989 until 2008, *I. scapularis* accounted for less than 1% of the total submissions, but after 2009, the abundance began to increase. By 2012 they accounted for 24.8% of ticks submitted to the Ohio Department of Health [30].

*Ixodes cookei* abundance was highest prior to 1990 but has since become very rare in our dataset. Yet, we know that the abundance of *I. cookei* in the Maine passive surveillance programme has been constant, even as *I. scapularis* submissions have increased [5]. It is possible that *I. cookei* abundance in PA has also remained relatively stable, but that we lack sufficient power to detect *I. cookei*. This inconsistency in submission rates may be due to the shift from free to per-submission charges for tick identifications that occurred in the mid-late 1990s.

The fourth and fifth most abundant tick species in our database were *R. sanguineus*, the brown dog tick (287 submission lots consisting of 332 specimens) and *A. americanum* (183 submission lots consisting of 196 specimens) (Table 1). Although *R. sanguineus* originated in Africa, it is a cosmopolitan urban pest species found worldwide in association with humans and their canine companions [31]. Snetsinger [7] suggested in 1968 that *R. sanguineus* had established breeding populations in Pennsylvania, but the abundance tapered off after 1968 and none exist in our database since 2002. In contrast, the submission rates of *A. americanum* increased and then leveled off from 1960 to 2000. Although not commonly encountered, we have specimens from as recent as 2016.

### Host associations

Vector-host associations (including host specificity) are important for predicting the risk of pathogen transmission and identifying key players in a sylvatic disease cycle. The specificity to host varies with the tick species. In our dataset generalist tick species (e.g. *D. variabilis*, *I. scapularis*, *I. cookei*) were found parasitizing a wide range of vertebrate hosts, while specialist species (e.g. *I. dentatus*, *I. marxi*, *I. muris*) were associated with a single host or limited to host size (e.g. small mammals or birds). This was consistent with other data in the literature.

Less commonly encountered tick species can sometimes lead to incorrect assumptions about host preferences and subsequent risk of pathogen transmission. While some tick species may be presumed to hold strict host preferences, they may bite humans if given the opportunity. For instance, *I. dentatus* bit humans in cabins that had been inhabited by their squirrel hosts in Maine and Vermont, and *I. cookei* was found on humans in West Virginia [32, 33]. *Ixodes texanus* was only found from raccoons in our dataset, but this species is known to feed on several mammalian hosts [24, 34, 35]. Commonly held misconceptions about host associations of certain tick species (e.g. *I. cookei* as “groundhog ticks”) based on lack of encounter may result in ignoring a potentially epidemiologically important vector (e.g. *I. cookei* is a vector of Powassan encephalitis virus and may potentially be another vector of *Ehrlichia muris* [36]).

### Multi-faceted approach to tick surveillance

Surveillance can be a powerful tool for the detection of introduced species (transient or established), emergent arthropod-borne pathogens, and disease risks due to increases or changes in vector community composition. Both passive and active surveillance strategies have their strengths and weaknesses but combined, they provide a more complete picture of tick community dynamics. Active tick surveillance approaches such as dragging, flagging, CO_2_-trapping, or live animal capture, can be very effective for assessing tick load by habitat [7, 24, 34, 37]. It can, however, be labor-intensive, costly, and difficult to implement over a wide geographical area. Passive surveillance is more cost-effective and less labor-intensive and can provide insight into ectoparasite abundance, host associations, or habitat associations across a wider geographical area [38]. Passive surveillance (particularly based on submissions by citizens) may run the risk of under-representing certain taxa or reflect a bias toward certain host associations. However, citizen-submitted tick collections can provide valuable baseline data on prevalence and likelihood of tick encounters and may be more strongly correlated with reported human cases of tick-borne diseases than active surveillance alone [39–42]. A community engagement programme that actively recruits ticks submitted by citizens should be coupled with support for a rigorously curated database of tick submissions.

Utilizing complementary strategies can help fill in knowledge gaps about tick prevalence. In a study using a combination of retrospective literature review, data compilation of specimens from archival collections, and active collection (dry ice, dragging and flagging) in counties presumed to be free of *A. americanum*, 68 of 77 counties of Oklahoma were determined to be colonized [41]. The metadata associated with a multi-pronged approach to tick surveillance (assuming proper data curation and management) can provide insight into tick-host associations, vegetation, seasonality, and shifts in population structure that can be used for modeling disease risk. Archival tick samples (or their DNA) can be useful for retrospective mining for research on the population genetics of ticks to detect gene flow, host shifts, or on their microbial inhabitants.

Implicit in any tick surveillance strategy is having trained tick biologists who can readily distinguish species by morphological and molecular characteristics. In the last 20 years, *I. scapularis* has become the most abundant tick species in Pennsylvania. While distinguishing *Ixodes* from other genera of ticks is fairly simple, species-level identification requires more detailed morphological examination, since there are six endemic species of *Ixodes* and three exotic species that could potentially be misidentified as *I. scapularis*. More generally, although many tick species are incompetent vectors of *B. burgdorferi*, they may be vectors and/or reservoirs of other pathogens/parasites, or acquire pathogens during co-feeding [24, 43–46]. It is therefore important to correctly identify tick species, not only for the determination of disease risk but also because the treatments for the pathogens they transmit may differ significantly.

## Conclusions

An ideal tick surveillance programme would not only utilize multiple approaches and have a dedicated tick biologist proficient at species identification on staff, but it would also take a proactive stance that is not limited strictly to immediate threats. Since 1993 (~25 years) there have been 28 publications on ticks from Pennsylvania, and 22 of them were focused on *I. scapularis* and/or the microbiota (mostly on pathogens) [23, 25, 46–71]. While *I. scapularis* is an important vector that warrants this attention, other tick species are being ignored. The consequences of neglecting other potential ticks of epidemiological significance include missing shifts in tick biodiversity, not identifying the potential causes of said shifts, not monitoring changes in range expansions of vectors, and not detecting the presence of introduced or established species. Recently, the presence of *H. longicornis* was reported in Pennsylvania, but we have no data on whether it has been introduced previously, or whether it has established populations. Given the potential risks that this parthenogenic tick poses (wide host range, possible vector of multiple pathogens, may induce meat allergies), this example highlights the potential dangers of focusing on only one vector-pathogen system [70–73]. Hybrid datasets from tick collections derived from multiple sources represent a powerful tool for mining past ecological and epidemiological events. Many states maintain county records on passive tick submissions to veterinary or medical health officials, but there may be other cryptic collections (and associated data) housed in museums, universities, government institutions, or with private individuals. Combining these data with other ectoparasite databases and currently unexplored collections will provide ectoparasite researchers the robust dataset needed for a massive meta-analysis. These cryptic ectoparasite collections will provide the basis for exploring hypotheses such as: (i) are shifts in tick populations correlated with increasing human encroachment on natural habitats; (ii) what are some phenological reasons for the increase in *I. scapularis* abundance; or (iii) if displacement of a dominant tick community species occurs, what are the implications for tick-borne disease risk? We anticipate that participating in such a study will fill in the gaps of knowledge about less-studied tick species as well as highlight the intrinsic value of museum collections of ectoparasites.

## Additional files

**Additional file 1: Figure S1.** Total population of Pennsylvania counties over time. Data were taken from the US Census for 1960, 1990, 2000, and 2010.

**Additional file 2: Table S1.** Specimens submitted from outside of the state of Pennsylvania.

**Additional file 3: Table S2.** Exotic specimens from known ranges outside PA, the continental USA, or North America.

**Additional file 4: Figure S2.** Dot-density map of all individual tick specimens across Pennsylvania from 1900 to 2017. Each point represents an individual tick specimen with its placement randomized within the county and each colored dot represents a different tick species.

**Additional file 5: Figure S3.** Presence or absence map of species with less than 150. Counties with zero submissions are dark, while the light areas represent the presence of one or more specimens for each genus.
