# Supplementary figures and images for: A 117-year retrospective analysis of Pennsylvania tick community dynamics
Source: Parasit Vectors. 2019 Apr 29;12:189. doi: 10.1186/s13071-019-3451-6 (PMC6489237; doi:10.1186/s13071-019-3451-6)

1960

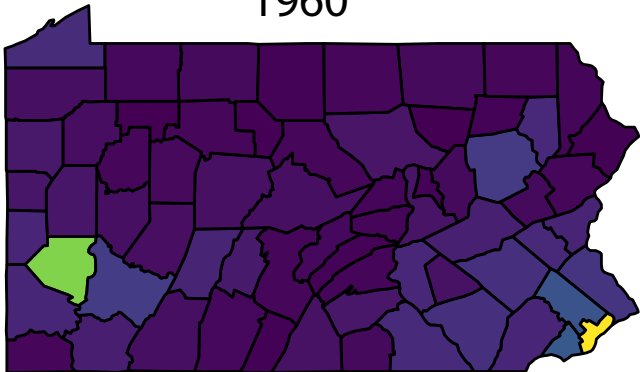

1990

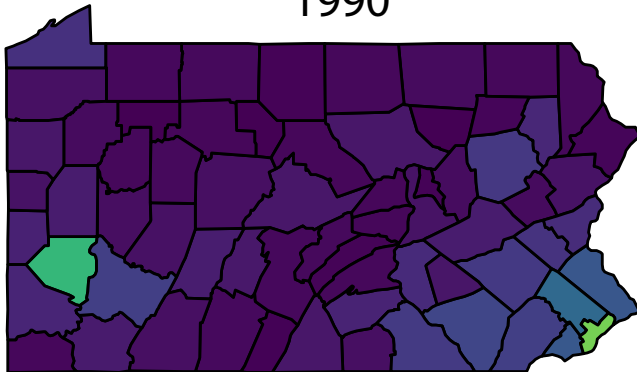

2000

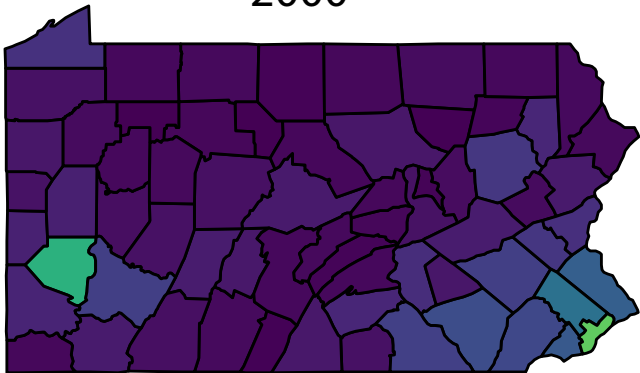

2010

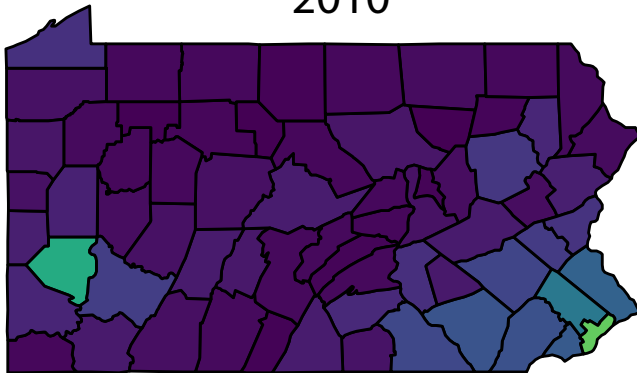

Population

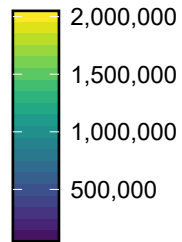

Supplement: Supplementary file 1 — Additional file 1: Figure S1. Total population of Pennsylvania counties over time. Data were taken from the US Census for 1960, 1990, 2000, and 2010. [file 13071_2019_3451_MOESM1_ESM.pdf]
