# Supplementary material for: A 117-year retrospective analysis of Pennsylvania tick community dynamics
Source: Parasit Vectors. 2019 Apr 29;12:189. doi: 10.1186/s13071-019-3451-6 (PMC6489237; doi:10.1186/s13071-019-3451-6)
Supplement: Supplementary file 2 — Additional file 2: Table S1. Specimens submitted from outside of the state of Pennsylvania. [file 13071_2019_3451_MOESM2_ESM.pdf]

Additional file 2: Table S1. Out-of-state specimens. Specimens declared from people visiting from or returning to other state.

Species	State	Submissions
Ixodes scapularis	North Carolina	1
Ixodes dentatus	Rhode Island	1
Amblyomma americanum	South Carolina	2
Amblyomma americanum	Tennessee	1
Dermacentor variabilis		1
Amblyomma americanum	Virginia	10
Dermacentor variabilis		1
Dermacentor variabilis	West Virginia	2
