# Supplementary material for: A 117-year retrospective analysis of Pennsylvania tick community dynamics
Source: Parasit Vectors. 2019 Apr 29;12:189. doi: 10.1186/s13071-019-3451-6 (PMC6489237; doi:10.1186/s13071-019-3451-6)
Supplement: Supplementary file 3 — Additional file 3: Table S2. Exotic specimens from known ranges outside PA, the continental USA, or North America. [file 13071_2019_3451_MOESM3_ESM.pdf]

Additional file 2: Table S2. Exotic tick specimens. Specimens that were not native to Pennsylvania, to northeastern United States, or to North America were likely introductions, either on exotic animals or other unknown sources [11]. Presence of exotic specimens may be indicative of introduction, but we do not have enough data to determine whether these species ever establish themselves as breeding populations. However, given the right conditions, an introduced exotic species can successfully establish a breeding population (for example, the parthenogenic *H. longicornis*) and become epidemiologically important to veterinary and human health.

**Species**	**Host**	**Native range**
Amblyomma cajennense	Capybara[1]	neotropics
Ixodes ricinus	Lizard[2]	palearctic
Amblyomma dissimile	Snake[3]	neotropics
Ambylomma maculatum	Human[4]	Southeastern USA
Argas persicus	Poultry[5]	Worldwide,<br>Southeastern USA
Argas cooleyi	Bat[5]	Western USA

[1] collected at the Philadelphia Zoological
[2] collected at the Pittsburgh Zoo
[3] collected Philadelphia.
[4] collected in Pennsylvania
[5] collected in Pennsylvania
[6] collected in Pennsylvania.
