# Supplementary material for: A 117-year retrospective analysis of Pennsylvania tick community dynamics
Source: Parasit Vectors. 2019 Apr 29;12:189. doi: 10.1186/s13071-019-3451-6 (PMC6489237; doi:10.1186/s13071-019-3451-6)

Tick submissions across Pennsylvania (1900−2017)

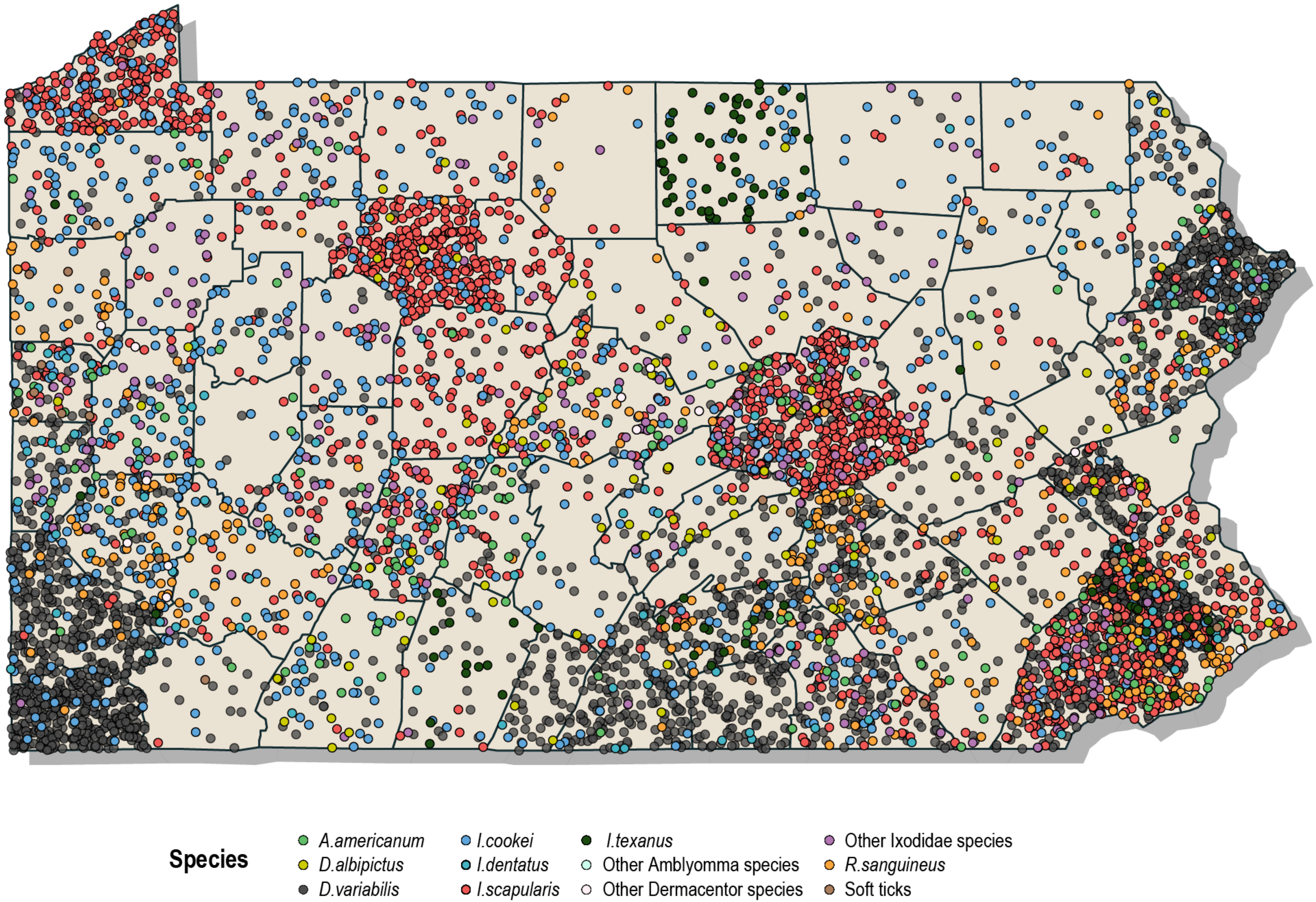

Supplement: Supplementary file 4 — Additional file 4: Figure S2. Dot-density map of all individual tick specimens across Pennsylvania from 1900 to 2017. Each point represents an individual tick specimen with its placement randomized within the county and each colored dot represents a different tick species. [file 13071_2019_3451_MOESM4_ESM.pdf]
