# Supplementary material for: A 117-year retrospective analysis of Pennsylvania tick community dynamics
Source: Parasit Vectors. 2019 Apr 29;12:189. doi: 10.1186/s13071-019-3451-6 (PMC6489237; doi:10.1186/s13071-019-3451-6)
Supplement: Supplementary file 5 — Additional file 5: Figure S3. Presence or absence map of species with less than 150. Counties with zero submissions are dark, while the light areas represent the presence of one or more specimens for each genus. [file 13071_2019_3451_MOESM5_ESM.pdf]

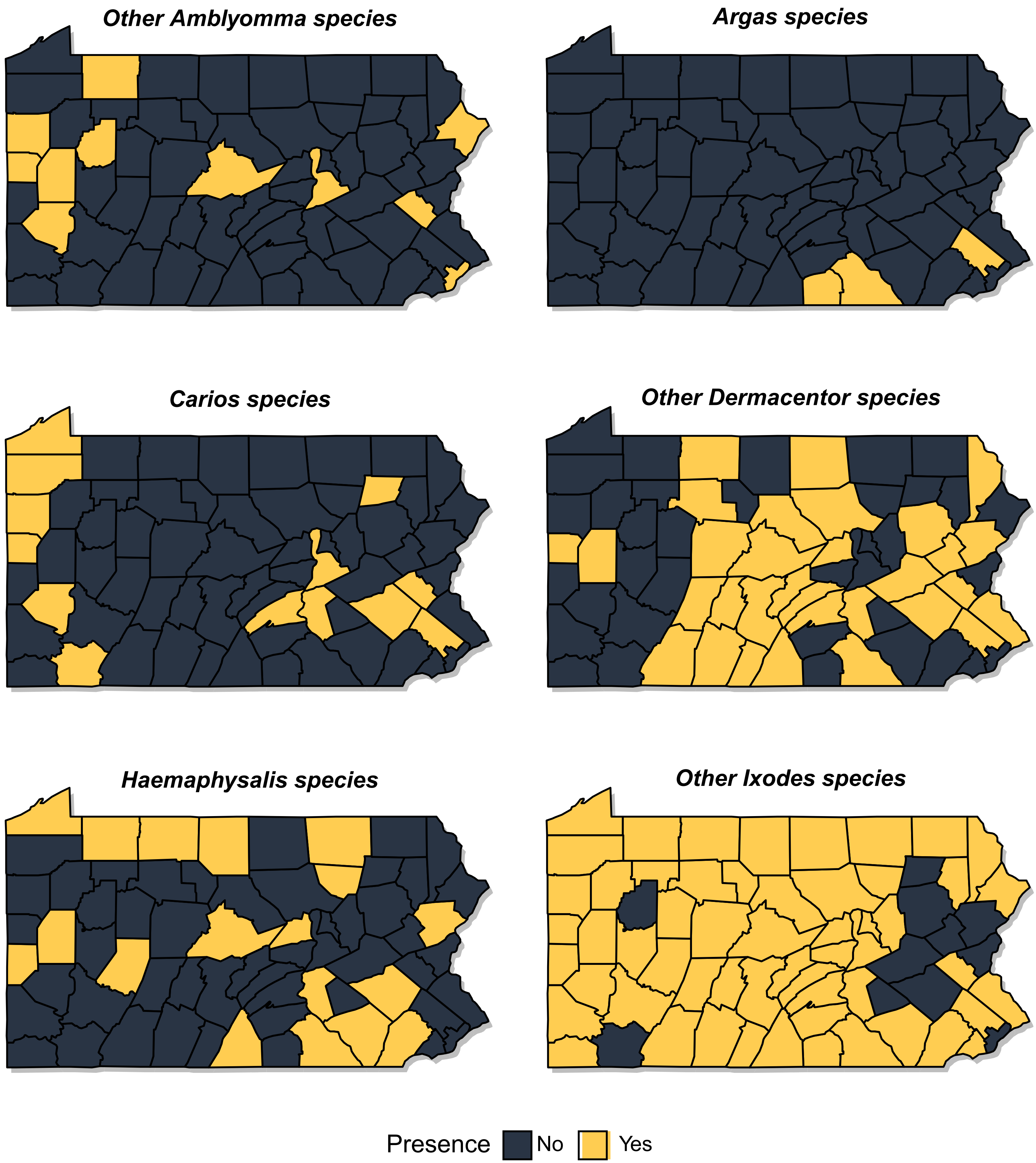

Other Amblyomma species
Argas species
Carios species
Other Dermacentor species
Haemaphysalis species
Other Ixodes species
Presence
No
Yes
